# Two Rings, Two Recoveries: Exploring the Thiersch Method in Complicated Cases

**DOI:** 10.7759/cureus.58818

**Published:** 2024-04-23

**Authors:** Aditya S Pedaprolu, Venkatesh M Rewale, Dheeraj P Surya, Sai Goutham Rekavari, Simran Dhole

**Affiliations:** 1 General Surgery, Datta Meghe Institute of Higher Education and Research, Wardha, IND

**Keywords:** anorectal disease, coloproctology, rectal surgery, thiersch procedure, procidentia, rectal prolapse

## Abstract

Rectal prolapse, characterized by the protrusion of rectal mucosa or full-thickness tissue through the anal canal, significantly impacts quality of life, necessitating prompt intervention upon diagnosis. This case report presents the management of rectal prolapse in two cases admitted to our institution presenting with complaints of a prolapsing anal mass and many associated comorbidities and their subsequent surgical management using the Thiersch procedure. Following the procedure, both patients were monitored postoperatively, discharged once vitally stable, and kept on regular follow-up. Surgery is the primary therapy for rectal prolapse, and while various surgical techniques address rectal prolapse, anal encirclement procedures like the Thiersch procedure have been largely replaced by newer abdominal or perineal approaches. However, it is a valuable option for select patient populations. The Thiersch procedure is an ideal modality for treating high-risk patients with rectal prolapse or those patients with poor compliance for more extensive procedures. It can also be used temporarily until a further definitive treatment is planned later in the future.

## Introduction

Rectal prolapse is defined as a protrusion of mucosa or full-thickness tissue of the rectal wall through the anal canal. It can be classified into two types: partial and complete (full thickness) prolapse. A partial or incomplete type involves prolapse of the mucosa only, and it may be circumferential or limited to only a portion of the rectal mucosa. By contrast, complete or full-thickness prolapse (procidentia) involves the whole bowel. Complete rectal prolapse is less common when compared with mucosal variety [1]. The precise cause of rectal prolapse is not fully understood, but certain factors seem to be implicated in its development. It is thought to commence as an intussusception of the rectum, which descends to protrude outside the anus. It usually occurs in persons at both extremes of life [2]. Medication-induced constipation in psychiatric patients and possible pelvic floor weakness in patients with previous pelvic surgery may be contributing factors to rectal prolapse. It is often associated with uterine prolapse in females. Symptoms include tenesmus (straining sensation), a sensation of mass protruding from the anus that may or may not spontaneously reduce, and a feeling of incomplete evacuation. Mucus discharge may accompany the protrusion. Patients also present with various functional complaints, from fecal incontinence (especially post-defecation) and diarrhea to constipation and outlet obstruction [3]. Physical examination with proctoscopy is a must and is essential to diagnose rectal prolapse and differentiate it from other prolapsing masses, such as cystocele, enterocele, or vaginal vault prolapse (seen in women). It is essential to note the descent of prolapse while straining or the Valsalva maneuver and the reducibility of the prolapsed mass.

Several radiological investigations, such as defecating proctography, magnetic resonance defecography, or dynamic perineal ultrasound, could aid in prolapse diagnosis. There have been numerous methods of management for rectal prolapse. Conservative management, which may be digital repositioning, pelvic floor exercises, submucosal injection, or banding, is done for infants and children. However, surgery is the primary therapy for rectal prolapse, and different procedures have been described to treat this condition. Operations can be categorized as either abdominal or perineal. The surgical treatment of rectal prolapse involves several operative techniques to correct the prolapse and address associated issues. These can be resective, fixative, or a combination of both to achieve anatomical repositioning of the bowel and improved function of the anorectal complex [4,5].

A few of these techniques are described as follows [6]: 1) The Moskowitz procedure involves reducing the prolapsed bowel and closing off the Douglas pouch. It was initially designed on the theory that the cause of rectal prolapse is a sliding hernia. 2) The Ripstein and Wells procedures involve securing the rectum to the sacral periosteum to prevent further prolapse. This procedure used an Ivalon sponge implant. It was enthusiastically popularized by the British and the Canadians. 3) The Frykman-Goldberg procedure combines sigmoid resection with rectopexy for redundant sigmoid colon cases. 4) Lahaut's operation, which implants a redundant sigmoid colon into the posterior rectal sheath. 5) Perineal procedures include the Delorme procedure, which involves mucosal proctectomy and muscular plication, and the Altemeier's procedure, which entails resecting the prolapsed rectum and sigmoid through the perineum. 6) Other perineal procedures include the Thiersch procedure for anal cerclage and the Gant procedure.

Carl Thiersch presented in 1891 a new method of surgical treatment of prolapse by insertion of a silver wire using a curve needle from the anus circumferentially around the anal canal. This intends to provide mechanical tension and strength of the tissues between the rectum and surrounding areas. Thiersch procedure (anal encirclement) is performed frequently in patients with old age or high risks with rectal prolapse. It is a simple procedure using a suture or prosthesis that narrows the anus when it was reported for the first time by Carl Thiersch. This minimally invasive procedure historically employed silver wire; the procedure now utilizes materials like Dacron, silastic, or silicone due to improved safety and preventing complications. It is a minor procedure indicated for patients with old age or hemodynamically unstable patients with existing comorbidities. Moreover, the Thiersch procedure would prevent strangulation and bleeding complications of an incarcerated rectal prolapse. However, this procedure has become obsolete due to postoperative complications of chronic peri-anal sepsis, anal stenosis, and obstructed defecation. In this instance, the Thiersch procedure indicated that due to the patient's senility and pre-existing cardiac and cerebrovascular complications, a minor procedure would be beneficial [7].

Out of all these procedures, Altemeier's procedure stands out for its versatility, being suitable for both electives and emergencies. In emergency cases, a diverting loop colostomy/ileostomy is often recommended. This procedure applies to patients of various ages and is particularly favorable for those with significant comorbidities. The choice of management strategy depends on several factors, such as age, gender, incontinence, comorbidities, prior prolapse repairs, physiologic testing, surgeon's experience, and, notably, preoperative constipation. Perineal approaches are more conservative, while abdominal approaches, which can be performed via laparotomy or laparoscopy, are more radical. These included Teflon, Marlex sling repair (Ripstein operation), or sutured posterior rectopexy [8]. The perineal approach is often preferred for elderly patients with severe comorbidities, while the abdominal approach is more suitable for younger patients with redundant sigmoid colon, constipation, or incontinence.

## Case presentation

Case 1

**Figure 1:**
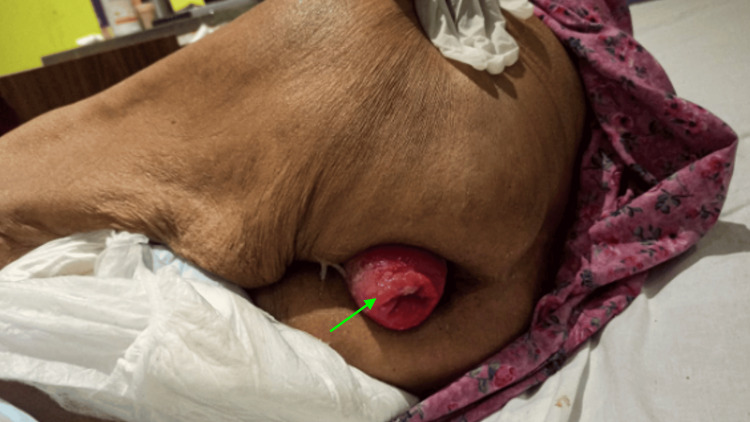
Case 1: preoperative presentation of prolapse (indicated by a green arrow).

A 75-year-old female presented to our outpatient department with complaints of a mass protruding from the anus (Figure 1).

**Figure 2:**
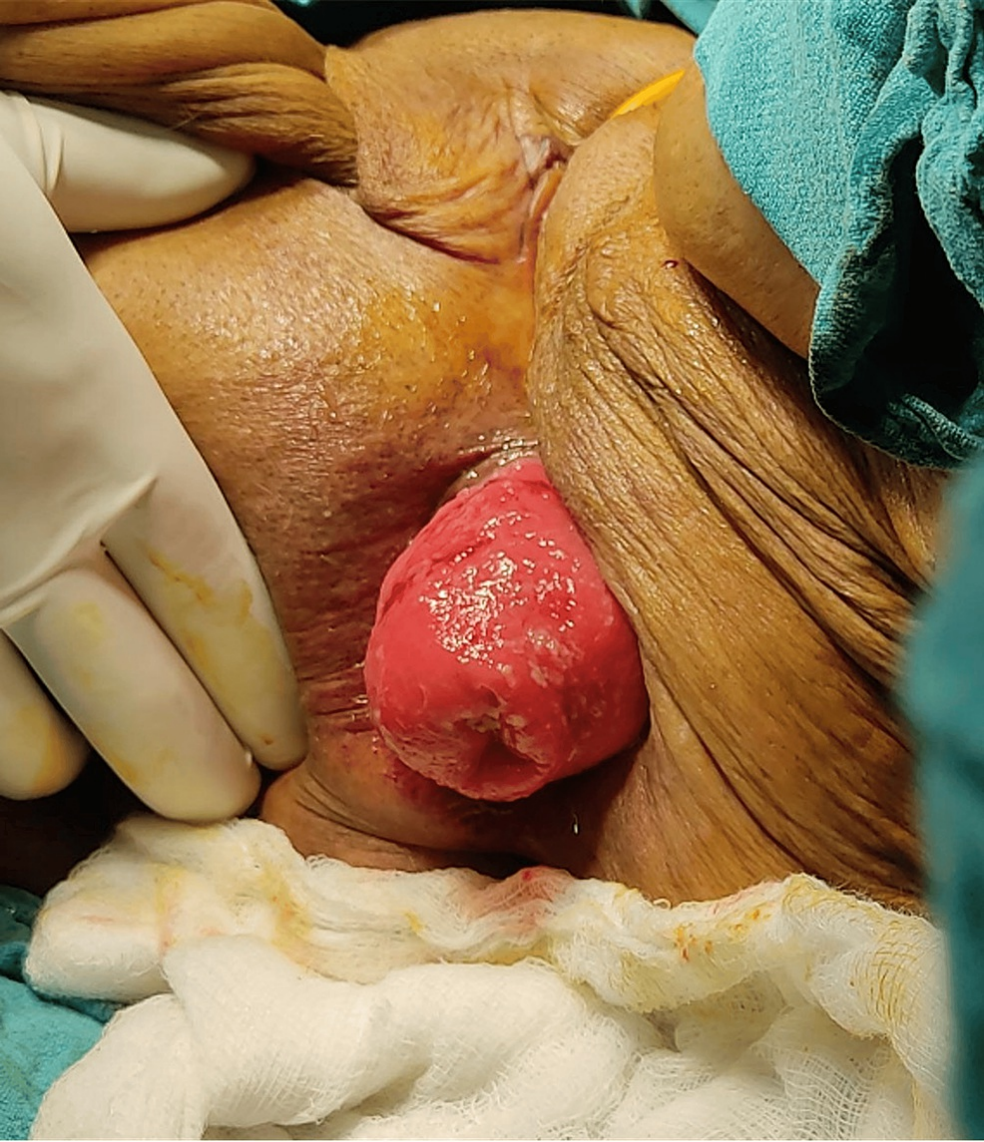
Case 1: intraoperative image. The rectal prolapse is everted by gentle traction on the rectal wall.

**Figure 3:**
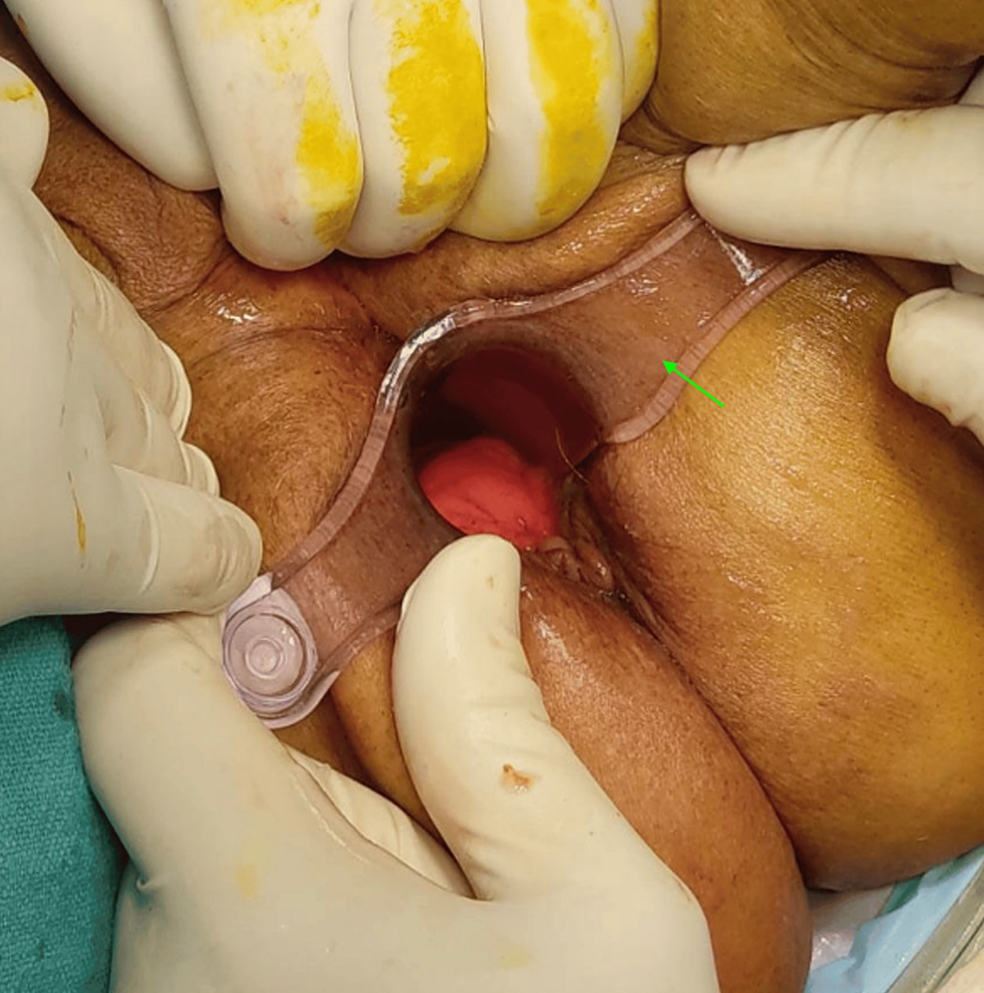
Case 1: insertion of the proctoscope (indicated using a green arrow).

**Figure 4:**
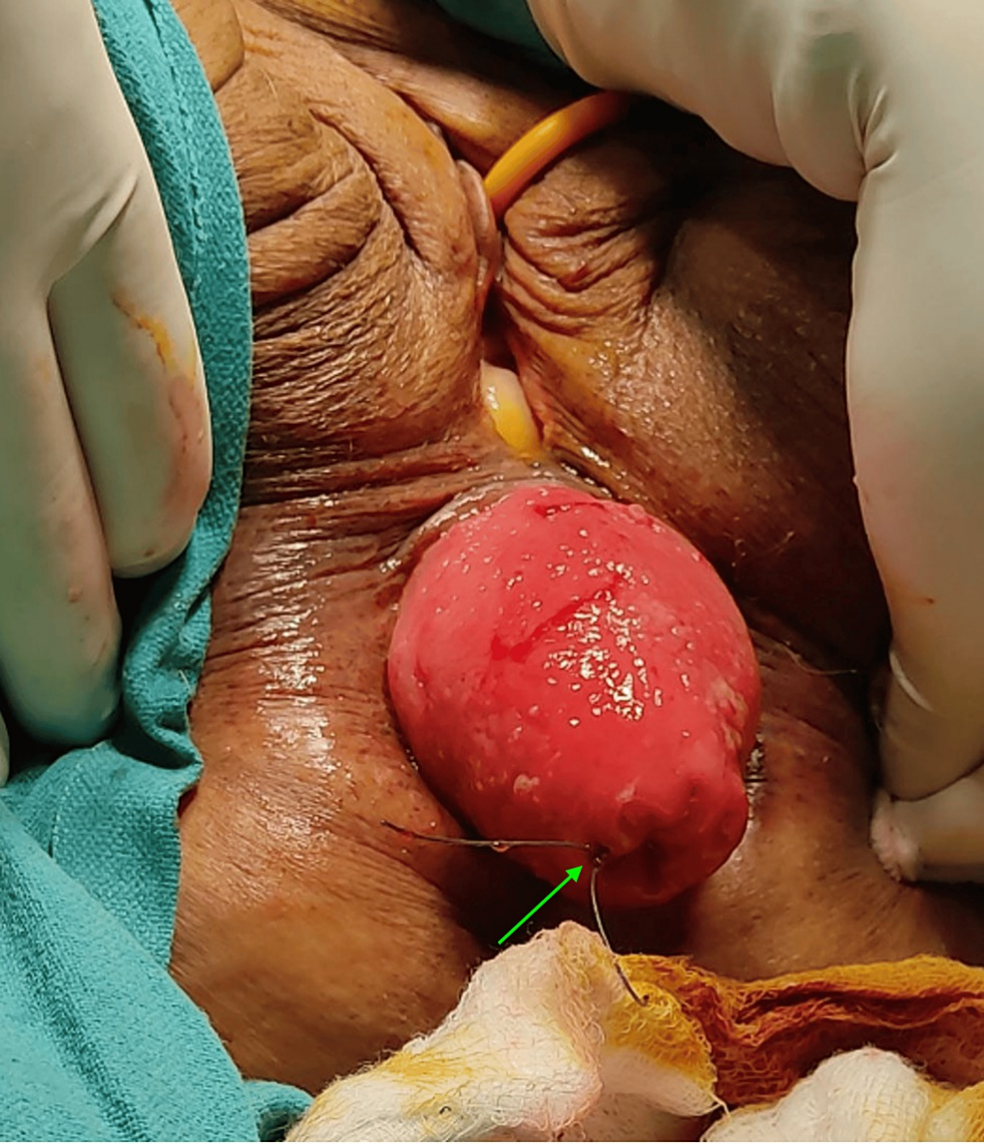
Case 1: intraoperative image. After injecting a sclerosant, a continuous interlocking suture (green arrow) was done using Prolene 1-0 (round body).

**Figure 5:**
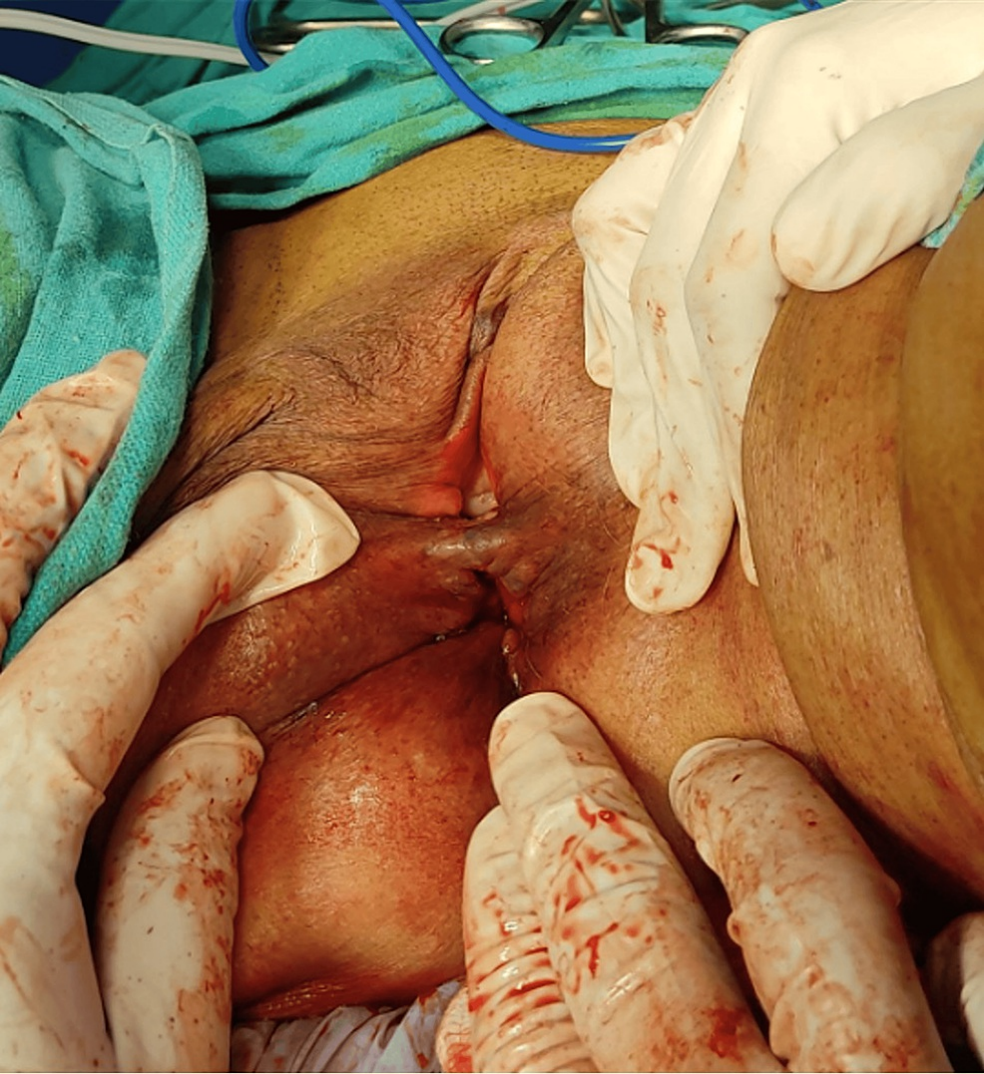
Case 1: postoperative image following the Thiersch procedure

At the same time, the patient also complained of proctalgia, along with bleeding per rectum and diarrhea. The patient is also a known case of congestive cardiac failure, severe aortic stenosis with anemia, and cystocele with a history of a cerebrovascular event in 2017 for which she is undergoing medical treatment. The patient has been hypertensive for 25 years and is currently on anti-hypertensive medication. The patient had no similar presentation in the past. Per rectal examination revealed a large protruding rectal mass with edema and a thickened congested wall. No active bleeding/ulceration was seen. The patient was planned for surgical management using the Thiersch procedure (Figures 2-5).

The rest of the postoperative course in the hospital was uneventful, and the patient was eventually discharged with regular followup for a period of 6 months. No signs of infection or complications like obstruction, incontinence, or recurrence were seen.

Case 2

**Figure 6:**
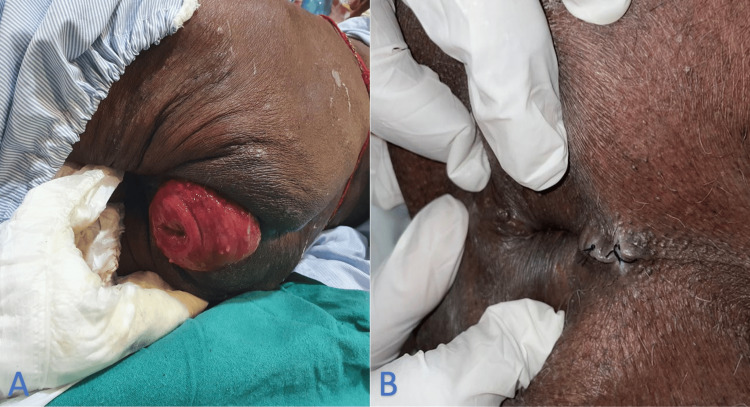
Case 2: preoperative clinical image (A) and postoperative image following the Thiersch procedure (B).

A similar case occurred in our hospital one year back where an 85-year-old male presented to our outpatient department with complaints of a protruding mass per rectum for five years associated with bleeding per rectum. He had known comorbidities of ischemic heart disease and hypertension for the past 15 years, for which he's on regular medication. He was subsequently operated on using the Thiersch procedure, and the procedure was uneventful (Figure 6). The indication for this procedure was also senility, underlying cardiac complications for which major surgery was contraindicated, and a suspected malignancy due to his chronic condition. A biopsy was taken and sent for histopathology intraoperatively, and the report was negative for any malignancy. The rest of the postoperative course in the hospital for this patient was also uneventful, and he was eventually discharged with regular followup. He developed no signs of infection, complications like obstruction, incontinence, or recurrence during followup.

## Discussion

There are several non-operative treatments for partial or complete rectal prolapse. However, it is only advised for infants or children or if a patient refuses surgery. These include adhesive strapping of buttocks and correction of constipation with laxatives, as demonstrated by Emma et al. [9]. In their study, she described the case of a three-year-old child with recurrent rectal prolapse, managed conservatively with laxative therapy. The child showed improvement over time with occasional recurrence [9]. Other conservative treatments include rubber ring ligation, sclerosing agent injection, or perineal strengthening exercises. However, these measures cannot be expected to produce a cure. Rectopexy is generally considered preferable by some surgeons for the correction of rectal prolapse, owing to the lower recurrence rates. However, in some instances, the Thiersch procedure can still be preferable even though it is obsolete. It can be carried out under a local anesthetic, making it a satisfactory technique for these high-risk individuals where sedation or general anesthesia cannot be given. It is a quick and minimally invasive procedure. It can also be used temporarily until definitive treatment is planned, as demonstrated by Naalla et al. [10]. In their case report, a 35-year-old man with complete rectal prolapse underwent a Thiersch procedure as a temporary measure due to hemodynamic instability. Once the patient's condition stabilized, he was subjected to posterior prosthetic rectopexy through an abdominal approach. The intention was to provide time for planning a more effective and permanent procedure [10].

Another standard minimally invasive procedure is injection sclerotherapy. Several sclerosants are used, most of which have been successful. Some of the substances used include alcohol, phenol, and reflux. Sclerotherapy using phenol-in-almond-oil injection has been reported to yield favorable results. Although safe, simple, and practical, phenol injection carries the risk of several possible complications, including bleeding, perirectal inflammation, urinary retention, ischiorectal abscess, and necrosis of rectal mucosa. If sclerotherapy fails, then the Thiersch procedure is recommended. The two procedures combined have a higher success rate than when performed separately, as demonstrated in the case report published by Chauhan et al. [11]. Here, three Thiersch sutures using PDS and proline were done in children suffering from possible Rasmussen's syndrome with recurrent complaints of rectal prolapse [11]. Other approaches have been advocated as alternatives or improvements to the Thiersch operation. Lomas and Cooperman have employed marlex mesh [8]. The use of a ribbon of Mersilene by Notaras, along with a deeper mesh placement, was practiced [7,12].

## Conclusions

Surgical procedures for managing rectal prolapse are diverse, and no explicit or precise gold-standard treatment strategy has been identified yet. Functional aspects and minimal invasiveness should be carefully considered during this process. Previously, the Thiersch procedure was done using primitive measures using steel wires, which led to many complications, making it obsolete. However, this procedure can still be a minor procedure for high-risk patients, controlling hemorrhage and a temporary treatment until a better procedure, if necessary, is planned. The Thiersch procedure is a valuable modality in treating high-risk patients with rectal prolapse. It can also be used temporarily until definitive treatment is planned. The surgical strategy depends on patient compliance, so a Thiersch procedure can be done on patients with poor compliance.
